# A Deep Learning System Using Optical Coherence Tomography Angiography to Detect Glaucoma and Anterior Ischemic Optic Neuropathy

**DOI:** 10.3390/jcm12020507

**Published:** 2023-01-07

**Authors:** Roxane Bunod, Mélanie Lubrano, Antoine Pirovano, Géraldine Chotard, Emmanuelle Brasnu, Sylvain Berlemont, Antoine Labbé, Edouard Augstburger, Christophe Baudouin

**Affiliations:** 1 Department of Ophthalmology 3, Quinze-Vingts National Ophthalmology Hospital, 28 Rue de Charenton, 75012 Paris, France; 2 Keen Eye Technologies SAS, 74 Rue du Faubourg Saint-Antoine, 75012 Paris, France; 3 Department of Ophthalmology 4, Quinze-Vingts National Ophthalmology Hospital, 28 Rue de Charenton, 75012 Paris, France; 4 IHU FOReSIGHT, INSERM-DGOS CIC 1423, 17 Rue Moreau, 75012 Paris, France; 5 INSERM, CNRS, Institut de la Vision, Sorbonne Universités, 7 Rue Moreau, 75012 Paris, France; 6 Department of Ophthalmology, Ambroise Paré Hospital, IHU FOReSIGHT, AP-HP, University of Paris Saclay, 9 Avenue Charles de Gaulle, 92100 Boulogne-Billancourt, France

**Keywords:** glaucoma, anterior ischemic optic neuropathy, optical coherence tomography angiography, artificial intelligence, deep learning, neuronal network

## Abstract

Introduction. Glaucoma and non-arteritic anterior ischemic optic neuropathy (NAION) are optic neuropathies that can both lead to irreversible blindness. Several studies have compared optical coherence tomography angiography (OCTA) findings in glaucoma and NAION in the presence of similar functional and structural damages with contradictory results. The goal of this study was to use a deep learning system to differentiate OCTA in glaucoma and NAION. Material and methods. Sixty eyes with glaucoma (including primary open angle glaucoma, angle-closure glaucoma, normal tension glaucoma, pigmentary glaucoma, pseudoexfoliative glaucoma and juvenile glaucoma), thirty eyes with atrophic NAION and forty control eyes (NC) were included. All patients underwent OCTA imaging and automatic segmentation was used to analyze the macular superficial capillary plexus (SCP) and the radial peripapillary capillary (RPC) plexus. We used the classic convolutional neural network (CNN) architecture of ResNet50. Attribution maps were obtained using the “Integrated Gradients” method. Results. The best performances were obtained with the SCP + RPC model achieving a mean area under the receiver operating characteristics curve (ROC AUC) of 0.94 (95% CI 0.92–0.96) for glaucoma, 0.90 (95% CI 0.86–0.94) for NAION and 0.96 (95% CI 0.96–0.97) for NC. Conclusion. This study shows that deep learning architecture can classify NAION, glaucoma and normal OCTA images with a good diagnostic performance and may outperform the specialist assessment.

## 1. Introduction

Glaucoma was the most common cause of irreversible blindness worldwide in 2020 and is predicted to become an increasing global health problem, with 111.8 million people expected to be suffering with glaucoma worldwide by 2040 [1,2]. Because glaucomatous retinal ganglion cell damage is irreparable, an early diagnosis and treatment is the key goal to limit permanent visual loss. However, the asymptomatic nature and slow progression of early visual field alterations explain why a considerable number of patients remain undiagnosed until a late stage of the disease [3]. The diagnosis of glaucoma is complex and based on a body of clinical parameters associated with structural and functional tests. Because of interindividual variations and variability in clinical tests, the challenge is to accurately detect early glaucoma or glaucoma progression while avoiding an overdiagnosis and overtreatment.

Artificial intelligence (AI) is the branch of computing science that aims to simulate human intelligence and has become one of the major technologic revolutions of the 21st century. AI has been extensively used in the health care industry for various purposes such as diagnosis, prognosis, drug development and patient care. Over the past decade, a subset of machine learning known as “deep learning” (DL) has emerged, providing an improved performance in computer vision challenges. This technology uses deep (multi-layer) neural networks (NN), modelled after the mammalian visual cortex, to analyze and extract patterns from complex image inputs. Since 2015, the number of approved AI-based devices has grown exponentially in the USA and Europe, with over half being applied in the field of radiology [4]. In ophthalmology, several AI approaches have been explored for the detection and diagnosis of various ophthalmologic diseases through imaging, such as retinal diseases (diabetic retinopathy, retinopathy of prematurity, retinal vein occlusion and retinal detachment), corneal pathologies, ocular cancer and glaucoma. In glaucoma, AI may represent a new tool for handling the challenges faced in clinical practice and in population-based screening strategies by more effectively identifying early glaucoma and its progression. Fundus photographs, optical coherence tomography (OCT) imaging and visual field tests have been widely used as the input data for both a glaucoma diagnosis and the detection of its progression with a high diagnostic performance [5]. DL algorithms trained on matched fundus and OCT images might even outperform human grading for the detection of glaucomatous eyes on fundus photographs [6].

While an elevated intraocular pressure (IOP) is the main treatable risk factor for glaucoma, it is now understood that vascular dysfunction is implicated in the pathogenesis of glaucoma [7]. Optical coherence tomography angiography (OCTA) provides a rapid, in vivo, qualitative and quantitative assessment of the retinal and optic nerve head (ONH) microcirculation using repeated structural B-scan OCTs to detect the motion contrast related to the blood flow. It has been widely used to assess microvascular damage in glaucoma with a good diagnostic ability and is now a part of routine ancillary testing for a glaucoma diagnosis and follow-up [8].

Non-arteritic anterior ischemic optic neuropathy (NAION) is another major cause of irreversible blindness or seriously impaired vision among patients over 50 years of age. The pathophysiology involves the transient hypoperfusion of the posterior ciliary arteries, leading to optic disc ischemia and edema [9]. Despite having different clinical presentations and pathophysiology, the differential diagnosis between glaucoma and NAION can be challenging in clinical practice for patients presenting in a late stage of the disease. However, an accurate diagnosis is essential to adapting the treatment and follow-up, as glaucoma is a progressive disease that requires a close monitoring and treatment, whereas NAION remains non-progressive in the atrophic stage.

Our group has reported a peripapillary and macular retinal capillary rarefaction in both NAION and glaucoma, with significant correlations between the vessel density (VD) and retinal nerve fiber layer (RNFL)’s thickness, the thickness of the ganglion cell complex (GCC) and the VF mean deviation (MD) [10,11,12,13]. Several studies have compared the OCTA findings in glaucoma and NAION in the presence of similar functional and structural damages, but sometimes with contradictory results. While significant lower radial peripapillary capillary (RPC) VD has been reported in open-angle glaucoma (OAG) and normal tension glaucoma (NTG) compared to NAION [14,15], other studies suggest the reverse [16] or no difference [17]. Regarding the macular microvasculature, comparable conclusions can be found in the literature with a greater loss in the macular superficial capillary plexus (SCP) VD in OAG compared to NAION [16,18]. The goal of the present study was to use a deep learning system to differentiate glaucoma and NAION on OCTA images of the macular and peripapillary plexus. In order to generalize the results to a wider range of cases, we included different types of glaucoma: OAG, angle-closure glaucoma, NTG, pigmentary glaucoma, pseudoexfoliative glaucoma and juvenile glaucoma.

## 2. Materials and Methods

### 2.1. Setting

This retrospective cross-sectional study was conducted at the Quinze-Vingts National Ophthalmology Hospital in Paris, France, between January 2019 and April 2021.

Patients and the study population. Patients with glaucoma and NAION followed at the Quinze-Vingts National Ophthalmology Hospital (Paris, France) were retrospectively included. Age-matched normal patients were recruited from the emergency department or from the patients attending physicians. Sixty eyes of 60 subjects with glaucoma were included. Among them were 36 eyes with primary open angle glaucoma (POAG), 13 eyes with angle-closure glaucoma, 2 eyes with normal tension glaucoma, 4 eyes with pigmentary glaucoma, 4 eyes with pseudoexfoliative glaucoma and 1 eye with juvenile glaucoma. In the NAION group, 30 eyes of 23 subjects (7 cases of bilateral NAION) were included. Among them, 17 patients had previously been included in another study by our group [11]. In the control group, 40 eyes of 40 normal subjects were included. The study was approved by our CPP-Ile-de-France Ethical Committee (number 10793) and adhered to the tenets of the Declaration of Helsinki. Written informed consent was obtained from all participants. A glaucoma diagnosis was made by a glaucoma specialist and defined as the presence of glaucomatous ONH changes (focal or diffuse neuroretinal rim thinning, RNFL and GCC defects on OCT) with compatible glaucomatous VF damage confirmed on >2 reliable VFs and a history of IOP > 21 mmHg (except in cases of normal tension glaucoma, for which a complementary work-up, including a brain MRI, carotid Dopplers, ECG, Holter blood pressure and complete cardiovascular examination, was performed). Glaucomatous VF loss was defined as a glaucoma hemifield test graded “outside normal limits” and a cluster of three contiguous points at the 5% level on the pattern deviation plot, using the threshold test strategy with the 24-2 test pattern of the Humphrey Field Analyzer II [19,20]. Different types of glaucoma were included: primary open-angle glaucoma, angle-closure glaucoma, pigmentary glaucoma, pseudoexfoliative glaucoma, juvenile glaucoma and normal tension glaucoma.

The inclusion criteria for NAION included a history of a sudden, painless loss of vision in patients over 40 years of age associated with optic disc edema, with or without superficial hemorrhage, and compatible visual field defects. All patients were referred to the internal medicine department to determine whether the diagnosis was arteritic or NAION based on a clinical examination, C-reactive protein level, erythrocyte sedimentation rate and temporal artery biopsy in uncertain cases. The data acquisition for the study was performed in the atrophic phase, at least 6 months after the diagnosis, after the complete resolution of the optic disc edema.

The control group included age-matched subjects with best-corrected visual acuity > 20/20, IOP < 21 mmHg, normal optic disc appearance on fundus examination, no GCC or RNFL defects on OCT and a normal VF.

The exclusion criteria for glaucoma, NAION patients and the control subjects were the presence of any significant systemic disorder, any associated maculopathy or neurological disease that may affect the optic disc or visual field, major media opacities or refractive errors >+6.00 or <−6.00 D.

### 2.2. Observation Procedure

For all patients, the following data were recorded: their age, sex, best corrected visual acuity (BCVA) on a logarithmic scale, Goldmann IOP (mmHg), mean RNFL thickness and mean GCC thickness (RTVue XR100 Avanti) and automated visual field (VF) mean deviation (Humphrey Visual Field Analyzer, SITA-Standard 24-2 program Carl Zeiss Meditec, Dublin, CA, USA). When both eyes of a subject were eligible, one eye was selected randomly, except for the NAION group, in which both eyes of the same patient were included in cases of bilateral NAION.

OCT-A imaging was performed using the RTVue XR100 Avanti with the AngioVue software (Optovue, Inc., Fremont, CA, USA) (details described in a previous study by our group [10]). All subjects underwent a 6 × 6 mm macular cube scan centered automatically on the fovea and a 4.5 × 4.5 mm cube scan centered automatically on the optic disc. Automatic segmentation by the AngioVue software (v2017.1.0.151) was used to analyze the vascular plexus. On macular OCT-A, the macular SCP and RPC were automatically defined, as described in our previous studies [10]. An eye tracker system for pupil movements was used during the image acquisition to minimize motion-related artifacts in addition to the Motion Correction Technology (MCT™). Images with an SSI < 45, aberrant segmentation or motion-related artifacts were excluded. For each patient, macular SCP and RPC in the face OCTA images were exported and anonymized.

The results were obtained using a stratified five-fold cross-validation method, dividing the dataset into a training set and an independent test set to compute the model’s performances. Classic data augmentation such as horizontal and vertical flip, rescaling and blur were applied to the training set to reduce overfitting. For this study, the classic convolutional neural network (CNN) architecture of ResNet50 [21] pre-trained on Imagenet [22] was combined with a fully connected layer. A softmax activation function was applied to the output vector to obtain a vector of probability (probability of belonging to a specific class). We measured the cross-entropy with regard to the ground truth label (loss) and adapted the model’s parameters with Stochastic Gradient Descent [23]. We used a method called Integrated Gradients to compute the attribution maps over testing samples that highlight the pixels that contributed most to the prediction [24]. Detailed and additional technical data can be found in Appendix A.

All of the OCT-A images were classified independently by two specialists in NAION (EA) and glaucoma (GC). The anonymized images of both SCP and RPC were presented randomly and analyzed by the specialists. Each image was classified based on the OCT-A images only. The ROC-AUC of the experts were computed considering the probability that the attributed class was equal to 1 and the others to 0. The KC, sensibility, specificity, accuracy and error rate were used to evaluate the expert’s performance.

### 2.3. Main Outcome Measure

The deep learning classifier performance was evaluated using the area under the receiver operating characteristics (AUC-ROC), sensitivity, specificity, accuracy and error rate. Cohen’s kappa coefficient (KC) was used to measure the level of agreement between the ground truth and the model’s predictions.

Data are presented as mean ± standard deviation or accompanied by 95% confidence intervals. BCVA was converted to the logarithm of the minimum angle of resolution (logMAR). The Mann–Whitney bilateral test was used for continuous data, and the Fisher exact test for categorical data. To compare the sensitivity and specificity, we used a bilateral McNemar test. The adjusted Wald method was used to compute 95% confidence intervals (95% CI) for sensitivities and specificities. *p* values < 0.05 were considered statistically significant. XLSTAT 2021 software was used for the statistical analysis.

## 3. Results

Sixty eyes were included in the glaucoma group, thirty eyes in the NAION group and forty eyes in the normal control group. There was no statistically significant difference in terms of the age between the three groups and no statistically significant difference in terms of the MD, average GCC thickness, average RNFL thickness between the glaucoma and AION groups (Table 1).

**Figure 1:**
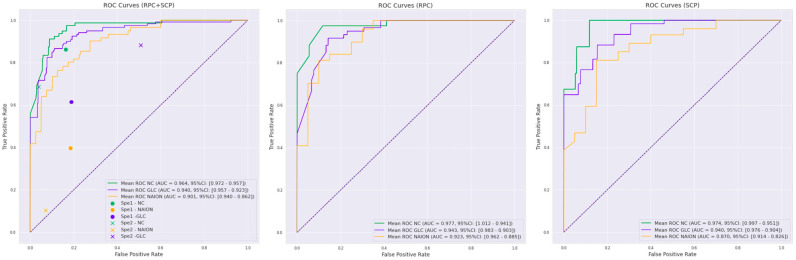
Receiver operating characteristics (ROC) curves for the three deep learning training protocols: mixed radial peripapillary capillaries plexus (RPC) + macular superficial capillary plexus (SCP) training, RPC training and SCP training. Normal control (NC, green curves), glaucoma (GLC, blue curves) and non-arteritic anterior ischemic optic neuropathy (NAION, yellow curves) corresponding to the mean ROC area under the curve (ROC-AUC) with a 95% confidence interval (95% CI) are indicated in the legend. Specialist 1 (Sp1) and specialist 2 (Sp2) performances are plotted on the RPC + SCP training curves for comparison.

The dataset was composed of 130 SCP images and 130 RPC images. For the mixed SCP and RPC model, the mean ROC AUC per class was 0.94 (95% CI 0.92–0.96) for glaucoma, 0.90 (95% CI 0.86–0.94) for NAION and 0.96 (95% CI 0.96–0.97) for NC (Figure 1). The KC between the ground truth and test set predictions was 0.69 (95% CI 0.63–0.75). For the RPC model, the mean ROC AUC per class was 0.94 (95% CI 0.90–0.98) for glaucoma, 0.92 (95% CI 0.89–0.96) for NAION and 0.98 (95% CI 0.94–1.0) for NC. (Figure 1).

The KC between the ground truth and test set predictions was 0.68 (95% CI 0.52–0.83). For the SCP model, the mean ROC AUC per class was 0.94 (95% CI 0.90–0.98) for glaucoma, 0.87 (95% CI 0.83–0.91) for NAION and 0.97 (95% CI 0.95–1.0) for NC (Figure 1). The KC between the ground truth and predictions was 0.60 (95% CI 0.58–0.60). The performances of the three models are summarized in Table 2.

The 260 anonymized mixed SCP and RPC images were independently classified by the two specialists. The specialists’ performance is summarized in Table 3 and plotted for comparison on the RPC + SCP training ROC curves (Figure 1).

For a glaucoma diagnosis, the deep learning model was statistically superior to both specialists in terms of the accuracy (0.87 95% CI 0.82–0.91 vs. 0.72 95% CI 0.67–0.78 and 0.67 95% CI 0.62–0.73) and error rate (0.14 95% CI 0.09–0.18 vs. 0.28 95% CI 0.22–0.33 and 0.33 95% CI 0.27–0.38) with non-overlapping 95% confidence intervals. This was also the case for an NAION diagnosis, with an accuracy of 0.86 95% CI 0.81–0.90 (vs. 0.72 IC 95% 0.67–0.78 and 0.75 95% CI 0.69–0.80) and an error rate of 0.14 95% CI 0.10–0.19 (vs. 0.28 95% CI 0.22–0.33 and 0.26 95% CI 0.20–0.31). A substantial agreement between the deep learning and the ground truth was observed (KC = 0.69 95% CI 0.63–0.75). On the other hand, a fair to moderate agreement was observed between the specialists’ classification and the ground truth (KC = 0.42 95% CI 0.35–0.48 and 0.40 95% CI 0.33–0.46). The McNemar test was performed to compare the sensitivity and specificity (Table 4). The model was significantly more sensitive for NAION (*p* = 0.04 and *p* < 0.001) and more specific for glaucoma (*p* = 0.03 and *p* < 0.001) than the two graders.

**Figure 2:**
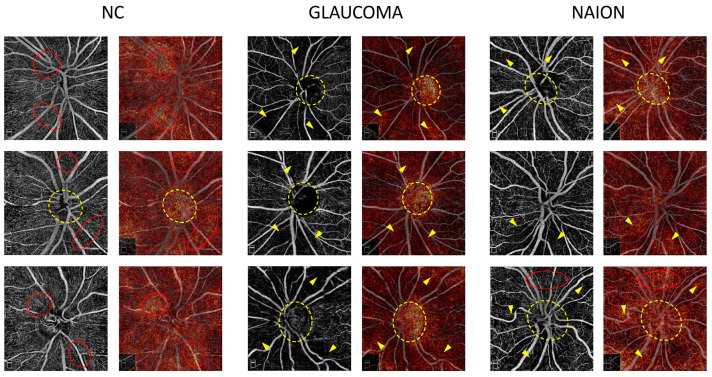
Examples of radial peripapillary capillaries plexus (RPC) attribution maps. RPC optical coherence tomography angiography (OCTA) for glaucoma, non-arteritic anterior ischemic optic neuropathy (NAION) and normal control (NC) correct attributions (**left column**) are represented with the corresponding attribution maps (**right column**). On the NC attribution maps, the most heavily highlighted areas correspond to the high vessel density areas on the OCTA image (red dotted circles) and in some cases to the optic nerve head (yellow dotted circle). On the glaucoma and NAION attribution maps, hot spots overlap the low vessel density areas (yellow arrows) on the OCTA image and the optic nerve head (yellow dotted circle). Areas with a preserved microvasculature (red dotted circle) can also be emphasized on the NAION attribution maps.

**Figure 3:**
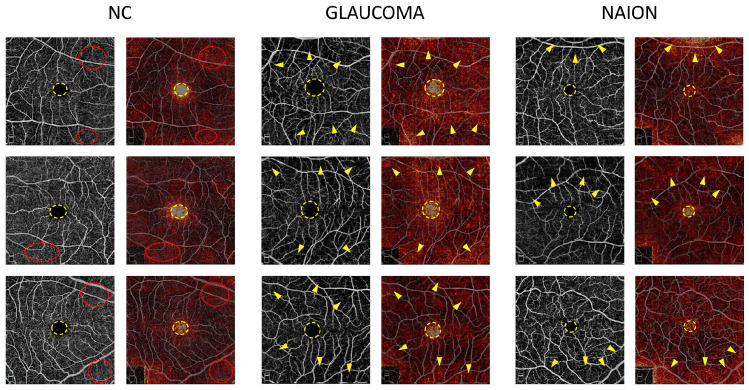
Examples of macular superficial capillary plexus (SCP) attribution maps. SCP optical coherence tomography angiography (OCTA) for glaucoma, non-arteritic anterior ischemic optic neuropathy (NAION) and normal control (NC) correct attributions (**left column**) are represented with the corresponding attribution maps (**right column**). In every case, the foveal avascular zone (FAZ) (dotted yellow circle) appears as a high spot. While the high vessel density areas (red dotted circles) are highlighted on the NC attribution maps, the low vessel density areas (yellow arrows) are emphasized on the glaucoma and NAION attribution maps.

Attribution maps were computed on the mixed SCP + RPC test set and were analyzed according to the prediction and ground truth. Figure 2 shows examples of the attribution map for glaucoma, NAION and NC on RPC images correctly classified by the model and Figure 3 shows examples of the attribution map for glaucoma, NAION and NC on SCP images correctly classified by the model.

## 4. Discussion

To our knowledge, this is the first study reporting the use of deep learning for an OCTA classification in optic neuropathies. We showed that the CNN is able to differentiate glaucoma, NAION and NC on macular and peripapillary OCTA images with an overall good sensitivity, specificity and substantial agreement. The performances were better with the mixed RCP + SCP training compared to RCP or SCP alone (Figure 1). On the other hand, RCP training demonstrated better results than SCP training alone. When comparing these results with the blinded specialists’ classification, deep learning architecture may outperform the clinical evaluation (KC 0.40 for specialist 1 and 0.45 for specialist 2 vs. 0.69 for RPC + SCP training).

Even for trained clinicians, the differential diagnosis between glaucoma and NAION can be difficult to make from a single view of the ONH if the clinical history is not clearly known or in a late stage of the disease. Indeed, optic disc pallor and/or cupping can be found in both pathologies, making the diagnosis challenging [25,26]. Glaucoma, due to its frequency and the large body of structural and functional imagery, is one of the pathologies for which deep-learning architecture applications may become very important, because any error or delay in the diagnosis can lead to irreversible visual loss. Several studies have shown good results of AI in differentiating glaucomatous changes from normal exams on fundus photographs, optical coherence tomography and standard automated perimetry [7]. However, in practice, it could also be useful for IA models to differentiate other common optic neuropathies.

Within a few years, OCTA has become an essential tool in neuro-ophthalmology. Through the detailed in vivo analysis of peripapillary capillaries and macular retinal plexuses, this technology has afforded an additional dimension in the understanding of optic neuropathies. Indeed, in the majority of studies, a decrease in the vessel density (VD) has been found even from early stages of the disease [8,11]. Moreover, the correlation between the decrease in the VD and the thinning of the RNFL and GCC layers has been demonstrated in both glaucoma and NAION [8,10].

Several studies have attempted to identify the differences between glaucoma and NAION from OCT-A images, sometimes with discordant results. Hondur et al. found a more significant loss of the peripapillary VD in the inferior and nasal sectors in POAG patients than in NAION subjects [14], whereas other authors did not [16,17]. Recently, Fard et al. found a significantly lower macular superficial VD and lower deep VD in the inferior and temporal parafoveal sectors in POAG compared to NAION eyes with the same GCC and RNFL thickness [18]. This would indicate specific areas of vulnerability to glaucomatous damage. Nevertheless, even knowing this, it seemed difficult for our two specialists to determine in each case the diagnosis associated with each OCTA image, on the one hand because there is a wide variety of sectorial involvement in both pathologies, and on the other hand because there is still no standard in OCTA devices that would allow for the rapid identification of a decrease in the capillary density in a specific sector. The response of the CNN, along with its confidence index, is additional information that may help the clinician.

Due to their multilayer nonlinear structure, NNs have been associated with the concept of a “black box” because of the lack of transparency and traceability of the predictions. Hence, several methods for interpreting deep NN results have been developed to show the connection between the input and output. A class activation map (CAM) display on a “heatmap” is the most valuable part of the image contributing to the prediction. We used an attribution method called “Integrated Gradients” [24] to build an attribution map on the test set RPC and SCP OCTA images. The other advantage of attribution maps is to provide information on the areas used by the CNN to reach these conclusions. The study of these attribution maps can either confirm the clinician’s analysis or direct attention to the unsuspected areas of the image.

On the RPC images of NC, the most highlighted pixels tend to be homogeneously spread across the scan, whereas in glaucoma and NAION, the inside of the optic disc is highlighted as the peripapillary area with a lower VD (Figure 2). We can speculate that the darker regions of the images associated with a VD loss in NAION and glaucoma are detected by the CNN and used to differentiate NAION and glaucoma from normal OCTA images. The inside-disc zone might be a region of interest in differentiating NAION from glaucoma, simply because ONH with a large excavation is mostly associated with glaucoma, while small ONH are more likely to be associated with NAION.

On the SCP attribution map, the FAZ was highlighted in glaucoma and NAION, as well as NC (Figure 3). To a lesser extent, areas with an SCP loss appear in the glaucoma and NAION attribution maps, whereas the overall image seems to appear in a uniform manner in NC. Similarly, in the RCP, the presence of darker regions might be used to differentiate glaucoma and NAION from a normal OCTA. At the center of the fovea, the FAZ is a round, capillary-free zone surrounded by a terminal ring of anastomotic capillaries originating from the superior and inferior temporal branches of the central retinal artery. OCTA has been shown to be highly reproducible in measuring the FAZ area in healthy patients, and an enlarged FAZ has been found in patients with a microcirculatory deficiency, such as diabetic retinopathy or vein occlusion [27,28,29]. Several studies have shown an enlarged FAZ area in OAG, angle-closure glaucoma, and NTG [30,31]. The association between the FAZ area and structural and functional damage has been assessed in previous studies. In a study by Igarashi et al., the FAZ area was significantly correlated with the RNFL thickness, GCC thickness, foveal threshold and MD value [32]. Li et al. showed that an enlarged FAZ area was associated with a higher risk of RNFL thinning in OAG [33]. To the best of our knowledge, there are no data available in the literature concerning the FAZ in NAION. However, in a previous study by our group, we noted that the perifoveal anastomotic ring appeared to be preserved in NAION [10]. Moreover, Liu et al. found no statistical difference in the parafoveal VD between normal and NAION patients [34]. Not only the area but also the shape and appearance of the surrounding “anastomotic circle” are known to be important parameters in retinal diseases. In glaucoma, the FAZ circularity index was one the most discriminating parameters in the study of Choi et al. [31]. We can speculate that CNNs are able to detect changes in the FAZ area and shape at the same time, as well as modifications to the terminal ring and parafoveal capillary network. The pattern of macular microvasculature alterations might be different in these two optic neuropathies, and further studies are required to compare the FAZ and parafoveal VD in glaucoma and NAION. According to the authors, while it is possible to predict the type of damage from the OCTA image of the RPC, it is very difficult to obtain from the macular images alone. The fact that AI is able to differentiate with a good accuracy the macular images from the three groups confirms its diagnostic utility.

The results of this study might provide an insight into the pathophysiological processes involved and the link between microvascular alterations and retinal ganglion cell loss. The underlying mechanisms are probably complex and different for each condition. In glaucoma, vascular studies have identified a low ocular perfusion pressure as a risk factor, but it is still debated if this is a cause or a consequence of RGC loss. Some studies have demonstrated that a reduction in the VD can precede glaucomatous structural alterations in both NTG and OAG, suggesting that a reduced ocular blood flow could be a primary event in glaucoma [35,36]. However, the link between IOP and microvascular alterations is still debated, and the results are controversial. Some studies have shown an FAZ and peripapillary reversal after a surgical or medical IOP reduction [37,38], whereas others found no change and persistent focal microvascular defects after the IOP normalization, which may result in longer term damage to the retina [13]. In this way, the mechanical and vascular theories may be linked, as elevated IOP may contribute to microvascular alterations. On the other hand, the topographic relationship between the decreased peripapillary microvasculature and RNFL defects, also found in other optic neuropathies, may suggest a reduced VD as secondary to RGC atrophy [39,40]. This idea is supported by histologic studies evidencing the close functional relationship between the RPC network and RGC axons [41]. In NAION, there is considerable evidence showing that a decreased VD is the consequence of the lower metabolic demand associated with RGC atrophy [42,43]. Similar vascular alterations have been found in other optic neuropathies, such as optic neuritis, suggesting that superficial VD alterations might be secondary to the RNFL damage [40,44,45].

The present study is subject to some limitations. Unfortunately, our dataset contained only a limited number of patients with an unbalanced group size due to the rarity of NAION and the single-center design of the study. However, we used a cross-validation method as a validation technique for assessing our model’s predictions and its ability to generalize to an independent dataset. This method uses a resampling process in order to avoid overfitting or a selection bias in a limited dataset. Moreover, the soundness of our results is backed up by narrow confidence intervals, reflecting the confidence level. However, even if the model’s performance is evaluated with various performance metrics, these metrics can be sensitive to a class imbalance. Deep capillary plexus (DCP) images were not always interpretable because of flow projection artifacts and automatic segmentation errors despite the projection of the artifact correction algorithm. Therefore, the DCP data were not included in the study. The inclusion of these images would be a way to improve the accuracy of the algorithm, as differences between the NAION and POAG subjects at the level of the DCP have been previously shown [18]. In this study, different types of glaucoma were included (POAG, angle-closure glaucoma, pigmentary glaucoma, pseudoexfoliative glaucoma, juvenile glaucoma and NTG). In a further study, it would be interesting to analyze separately the different types of glaucoma to assess if the different pathogenetic mechanisms may affect the diagnostic performances. While interpreting the attribution maps, it is important to consider that the choice of the baseline used in the integrated gradient method and the interpretation of the resulting attribution map remain subjective and can be subject to an interpretation bias. AI analyses are expected to grow in the future through the development of multi-center databases to provide an increasing number of images and thus improve the accuracy of deep learning architecture. We can also speculate that the combination of the various structural and functional exams and measurable clinical data will also be a way to improve the performance of AI algorithms. In this process, OCTA imaging is expected to constitute an additional element for AI training in the future.

## 5. Conclusions

In conclusion, this study is the first to use a deep learning algorithm to classify normal control subjects, NAION and glaucoma based on OCTA images. The diagnostic performance was good and outperformed the specialists’ assessment. The analysis of the attribution map highlighted the importance of the FAZ and inside-disc VD, though further studies are required to explore and compare these areas in glaucoma and NAION.

## Figures and Tables

**Table 1:** Demographic and ophthalmic characteristics.

	Glaucoma (*n* = 60)	NAION (*n* = 30)	NC (*n* = 40)	Glaucoma vs. NAION (*p*)	Glaucoma vs. NC (*p*)	NAION vs. NC (*p*)
Demographic characteristics						
Mean age, years	63.2 ± 13.3	67.6 ± 8.7	62.5 ± 11.4	0.173	0.858	0.132
Female/male	21/39	5/25	27/13	0.135	0.002 *	<0.001 *
High blood pressure, %	26.7	55.2	32.5	0.011 *	0.653	0.084
Diabetes, %	20.0	20.7	10.0	1.00	0.266	0.302
Ischemic heart diseases, %	5.0	10.3	7.5	0.387	0.681	0.690
OSAS, %	5.0	20.7	2.5	0.054	0.648	0.036 *
Ophthalmic characteristics						
BCVA, logMar	0.21 ± 0.39	0.63 ± 0.71	0.02 ± 0.05	<0.001 *	<0.001 *	<0.001 *
IOP, mmHg	16.3 ± 5.7	14.7 ± 3.3	14.4 ± 3.7	0.329	0.134	0.484
RNFL thickness, μm	63.23 ± 10.87	70.00 ± 16.04	98.25 ± 9.29	0.058	<0.001 *	<0.001 *
GCC thickness, μm	67.05 ± 10.04	67.79 ± 12.73	97.28 ± 6.07	0.823	<0.001 *	<0.001 *
Visual Field MD, dB	−18.21 ± 7.86	−20.46 ± 6.94	0.10 ± 1.6	0.164	<0.001 *	<0.001 *

NAION: nonarteritic anterior ischemic optic neuropathy; NC: normal control; OSAS: obstructive sleep apnea syndrome; BCVA: best corrected visual acuity; IOP: intra-ocular pressure; RNFL: retinal nerve fiber layer; GCC: ganglion cell complex; MD: mean deviation; *p*: *p*-value. * Statistically significant result.

**Table 2:** Performance of the deep learning architecture for glaucoma, non-arteritic anterior ischemic optic neuropathy and normal control optical coherence tomography angiography (OCTA) classification.

		Sensitivity	Specificity	Accuracy	Error Rate	ROC-AUC	*k*
RPC	Glaucoma	0.90 (0.79–0.96)	0.80 (0.69–0.88)	0.85(0.78–0.91)	0.15 (0.09–0.22)	0.94 (0.90–0.98)	0.68 (0.52–0.82)
	NAION	0.53 (0.36–0.70)	0.94 (0.87–0.97)	0.85 (0.78–0.91)	0.15 (0.09–0.22)	0.92 (0.89–0.96)	
	NC	0.85 (0.70–0.93)	0.93 (0.86–0.97)	0.91 (0.86–0.96)	0.09 (0.04–0.14)	0.98 (0.94–1.0)	
SCP	Glaucoma	0.85 (0.74–0.92)	0.82 (0.71–0.90)	0.84 (0.77–0.90)	0.17 (0.10–0.23)	0.94 (0.90–0.98)	0.60 (0.58–0.61)
	NAION	0.67 (0.48–0.81)	0.84 (0.75–0.90)	0.80 (0.73–0.87)	0.20 (0.13–0.27)	0.87 (0.83–0.91)	
	NC	0.65(0.50–0.78)	0.95 (0.88–0.99)	0.86 (0.80–0.92)	0.14 (0.08–0.20)	0.97 (0.95–1.0)	
RPC + SCP	Glaucoma	0.83 (0.75–0.88)	0.90 (0.83–0.93)	0.87 (0.82–0.91)	0.14 (0.09–0.18)	0.94 (0.92–0.96)	0.69 (0.63–0.75)
	NAION	0.59 (0.46–0.70)	0.93 (0.89–0.96)	0.86 (0.81–0.90)	0.14 (0.10–0.19)	0.90 (0.86–0.94)	
	NC	0.92 (0.84–0.97)	0.87 (0.81–0.91)	0.88 (0.84–0.92)	0.12 (0.08–0.16)	0.96 (0.96–0.97)	

Data are given as mean (95% confidence interval). RPC: radial peripapillary capillary plexus; SCP: macular superficial capillary plexus; NAION: nonarteritic anterior ischemic optic neuropathy; NC: normal control; ROC-AUC: area under the receiver operating characteristics curves; *k*, Cohen’s kappa coefficient.

**Table 3:** Performance of specialists for glaucoma, non-arteritic anterior ischemic optic neuropathy and normal control optical coherence tomography angiography (OCTA) classification.

		Sensitivity	Specificity	Accuracy	Error Rate	*k*
Specialist 1	Glaucoma	0.62 (0.53–0.70)	0.81 (0.74–0.87)	0.72 (0.67–0.78)	0.28 (0.22–0.33)	0.42 (0.35–0.48)
	NAION	0.40 (0.28–0.53)	0.82 (0.76–0.86)	0.72 (0.67–0.78)	0.28 (0.22–0.33)	
	NC	0.86 (0.77–0.92)	0.84 (0.78–0.88)	0.85 (0.80–0.89)	0.16 (0.11–0.20)	
Specialist 2	Glaucoma	0.88 (0.81–0.93)	0.49 (0.41–0.58)	0.67 (0.62–0.73)	0.33 (0.27–0.38)	0.40 (0.33–0.46)
	NAION	0.10 (0.05–0.21)	0.93 (0.89–0.96)	0.75 (0.69–0.80)	0.26 (0.20–0.31)	
	NC	0.69 (0.58–0.78)	0.96 (0.92–0.98)	0.88 (0.84–0.92)	0.12 (0.08–0.16)	

Data are given as proportions (95% confidence interval). NAION: nonarteritic anterior ischemic optic neuropathy; NC: normal control; *k*, Cohen’s kappa coefficient.

**Table 4:** Comparison of sensitivity and specificity between deep learning classifier and specialists’ classification using the bilateral McNemar test.

	Specialist 1	Specialist 2	RPC + SCP	Sp1 vs. RCP + SCP (*p*)	Sp2 vs. RPC + SCP (*p*)
Sensitivity					
Glaucoma	0.62	0.88	0.83	<0.001 *	0.248
NAION	0.40	0.10	0.59	0.04 *	<0.001 *
NC	0.86	0.69	0.92	0.267	<0.001 *
Specificity					
Glaucoma	0.81	0.49	0.90	0.029 *	<0.001 *
NAION	0.81	0.93	0.93	0.001 *	1.00
NC	0.83	0.96	0.86	0.473	<0.001 *

*p*: Bilateral McNemar’s test *p*-value. Sp1: specialist 1; Sp2: specialist 2; RCP + SCP: radial peripapillary capillary plexus + macular superficial capillary plexus training; NAION: non-arteritic anterior ischemic optic neuropathy; NC: normal control. * Statistically significant result.

## Data Availability

The data presented in this study are available on request from the corresponding author.

## Appendix A

Dataset preparation

Each patient from our dataset belongs to one unique class. As patients were recruited over a period of years, images were acquired with two different resolutions (400 pixels × 400 pixels and 304 × 304 pixels). All images were resized to the same pixel resolution of 304 × 304 pixels.

Cross-validation

The dataset was split five-fold. During one cross-validation iteration, four splits of the dataset were used to train the model and perform early stopping (training set and validation set), and the remaining split was used to evaluate its performance (test set). The splits were performed in a stratified way to maintain the proportion of the images from each class across the splits. Stratified splits were obtained with the Scikit-Learn python library. Since the dataset could contain several images from one patient (SCP and RPC images in the “SCP + RPC” training, two eyes for some patients in the NAION group), images from a single patient were attributed to the same split.

Network architecture

We used the neuronal network ResNet50 as the model has proven to be highly effective for image classification tasks. It was the winner of the ImageNet Large Scale Visual Recognition Challenge (ILSVRC) in 2015 and has since become a widely used benchmark for image classification tasks. The ResNet50 model is relatively lightweight and efficient, allowing it to train several models relatively fast (especially for cross-validation). The model is easy to implement, with pre-trained weights and models available in many deep learning libraries like Tensorflow. The use of pre-train weights allows it to reach higher performances, even with a restricted dataset (like in our case). The ResNet50 model has been widely tested and validated on a variety of image classification tasks. It is well known by the computer vision community, allowing us to easily compare models as well as understand and interpret the results. Finally, the ResNet50 model has a relatively shallow architecture, making the interpretation and gradient-based heatmap visualization more understandable. Four blocks of ResNet 5021 pre-trained on Imagenet [22] were used to average the pooling with a fully connected layer added on top. Because we used Imagenet pre-trained weights to initialize the network, images were then rescaled to 224 × 224 pixels, and the unique channel was duplicated three times to obtain the final shape of the 224 × 224 × 3 matching Imagenet pre-training inputs. The images were normalized using the Imagenet mean per channel and standard deviation per channel. The output (dense) layer had three neurons for the three classes classification (glaucoma-NAION-NC).

CNN training

We measured the cross-entropy with regard to the ground truth label (loss) and adapted the model’s parameters with a stochastic optimization algorithm (ADAM optimizer, learning rate of 0.01). ADAM is one of the most common optimizers; unlike some other optimization algorithms, it typically requires little hyperparameter tuning and can often be used with default settings, limiting the possibility of overfitting, especially in the context of a small dataset. In addition, ADAM can adaptively adjust the learning rate, leading to overall good performances. The learning rate was set by testing a set of values in the first split of data. An L2 regularization term was added to mitigate the overfitting. We used a batch size of 8.

Formula for cross-entropy:

$$CE\left(P,G\right)=-\sum_{i}^{N}Gi\times \log\left(Pi\right)$$

*N* = number of classes; *P* = predicted values; *G* = ground truth one-hot label.

To avoid overfitting, an early stopping strategy was used: every 100 iterations, the average loss on the validation set was measured. The training was stopped, and the best model was saved if the average validation loss did not decrease after 2000 iterations (patience of 20 rounds).

Testing and performance metric

The results provided correspond to the average performances of our models (trained five times on five different splits of the dataset).

We computed various metrics to measure the performance of the model. The area under the receiver operating characteristics curve (ROC-AUC) was used to evaluate the classifier output quality. The multi-class ROC-AUC were obtained by binarizing the classes, in a one-vs.-all manner. Cohen’s kappa coefficient (KC) was used to measure the level of agreement. Other metrics were obtained using traditional true positive (TP), true negative (TN), false positive (FP) and false negative (FN) numbers computed for each class, each class being binarized in a one-vs.-all manner: recall = TP/(TP + FN); specificity = TN/(TN + FP); overall accuracy = (TP + TN)/(TP + FP + FN + TN).

Attribution maps

The Integrated Gradients method [24] was used to compute the attribution maps. For model F, the attribution map Am is computed as:

$$Am\left(xi;F,x^{\prime }\right)=\left(xi-x^{\prime }i\right).\sum_{k=0}^{m}\frac{\delta F(x^{\prime }+\frac{k}{m}\cdot \left(x-x^{\prime }\right))}{\delta xi}\cdot \frac{1}{m}$$
